# Hidden Impact of Hemodialysis on Informal Caregivers: A Cross-Sectional Study of Caregiver Burden in North India

**DOI:** 10.7759/cureus.116962

**Published:** 2026-09-26

**Authors:** Sana Shadab, Kaushalendra N Tripathi, Arvind Gupta, Santosh K Maurya, Abhishek P Singh, Abdullah

**Affiliations:** 1 Nephrology, Institute of Medical Sciences, Banaras Hindu University, Varanasi, IND; 2 Nephrology, Motilal Nehru Medical College, Prayagraj, IND; 3 Medicine, Motilal Nehru Medical College, Prayagraj, IND; 4 Psychiatry, Motilal Nehru Medical College, Prayagraj, IND

**Keywords:** caregiver burden, caregiver strain, chronic kidney disease, dialysis, hemodialysis, india, predictors, zarit burden interview

## Abstract

Introduction: Caregivers of patients on maintenance hemodialysis experience significant physical, emotional, and financial challenges that can profoundly affect their personal lives. This study aimed to assess caregiver burden and its associated factors among the caregivers of hemodialysis patients.

Methods: This analytical cross-sectional study was conducted at a tertiary care teaching hospital in northern India over a three-month period in 2026. Caregiver burden was assessed using the validated 12-item Zarit Burden Interview (ZBI-12), and logistic regression was used to evaluate its association with sociodemographic and clinical factors.

Results: A total of 130 caregivers were enrolled in the study. The mean age was 36.85 ± 12.98 years, and most were males (n=90, 69.2%). The majority of caregivers were spouses (n=42; 32.3%). Among them, 45.4% (n=59) were graduates or had higher education, and 52.3% (n=68) were employed. The median dialysis vintage was 10 months (IQR, 6-20). The mean caregiving duration was 5.46 ± 1.52 hours per day. The mean ZBI-12 score was 22.32 ± 7.21, and 77 caregivers (59.2%) reported a high caregiver burden (ZBI-12 score >20). Lower socioeconomic status, defined as the combined lower and upper-lower classes of the modified Kuppuswamy scale (2025), was independently associated with high caregiver burden in multivariable analysis (adjusted OR 2.90, 95% CI 1.29-6.52; p=0.010).

Conclusion: Caregiver burden is highly prevalent among informal caregivers of hemodialysis patients, with a higher burden observed among those from lower socioeconomic groups. Routine screening for caregiver burden and targeted caregiver education, particularly for resource-constrained households, may help to reduce caregiver strain.

## Introduction

The global prevalence of chronic kidney disease (CKD) is substantial [1], with a disproportionate burden in low- and middle-income countries (LMICs) [2]. In India, the estimated burden of CKD and end-stage kidney disease (ESKD) is approximately 800 and 200 per million population, respectively [3]. As the burden of chronic diseases increases, so does the need for informal caregivers [4,5]. In addition to health care workers, informal caregivers also play a pivotal role in managing hemodialysis patients. These informal caregivers are mostly family members who dedicate themselves to caring for their loved ones. Beyond emotional and financial support, they also provide direct assistance with disease care and activities of daily living, as well as transportation to dialysis units [6].

Caregiver burden is a major public health concern, and these informal caregivers may actually be "hidden patients" whose physical, psychological, and financial well-being are adversely impacted. It refers to the extent to which caregivers perceive that caring for a relative negatively impacts their emotional well-being, physical health, social life, and financial status [7]. Caregiver burden is influenced by various socioeconomic, demographic, and patient-related clinical factors [8]. The prevalence of moderate to severe caregiver burden among informal caregivers of hemodialysis patients ranges from 12% to 88% across studies [9-15] because of differences in study populations, assessment tools, and burden thresholds. However, the data on caregiver burden, particularly within the distinct sociodemographic context of northern India, remain limited.

This study aimed to assess caregiver burden using the 12-item Zarit Burden Interview (ZBI-12) and identify caregiver- and patient-related factors associated with high caregiver burden (ZBI-12 score >20) among informal caregivers of patients undergoing maintenance hemodialysis and attending the Nephrology OutPatient Department (OPD) at a tertiary care teaching hospital in northern India.

## Materials and methods

This analytical cross-sectional study was conducted in the Department of Nephrology at the Motilal Nehru Medical College, a tertiary-care teaching hospital in Uttar Pradesh, northern India, over a three-month period in 2026. This hospital serves the rural and urban population of Prayagraj and the adjoining districts of Pratapgarh, Jaunpur, Bhadohi, Mirzapur, and Chitrakoot.

Participants were recruited from the Nephrology OPD. Patients undergoing maintenance hemodialysis for more than three months at our dialysis unit or localized government dialysis units and receiving regular clinical follow-up at our center were screened along with their primary informal caregivers. Patients with significant comorbid conditions, including cardiac, neurological, pulmonary, hepatic, or psychiatric diseases, as well as malignancy requiring additional care, were excluded to assess the specific impact of dialysis-related care on caregiver burden. Caregivers were eligible if they were aged 18 years or older, had personally provided care to the patient for more than three months, spent the greatest proportion of time providing care, and provided written informed consent to participate (Figure 1).

**Figure 1:**
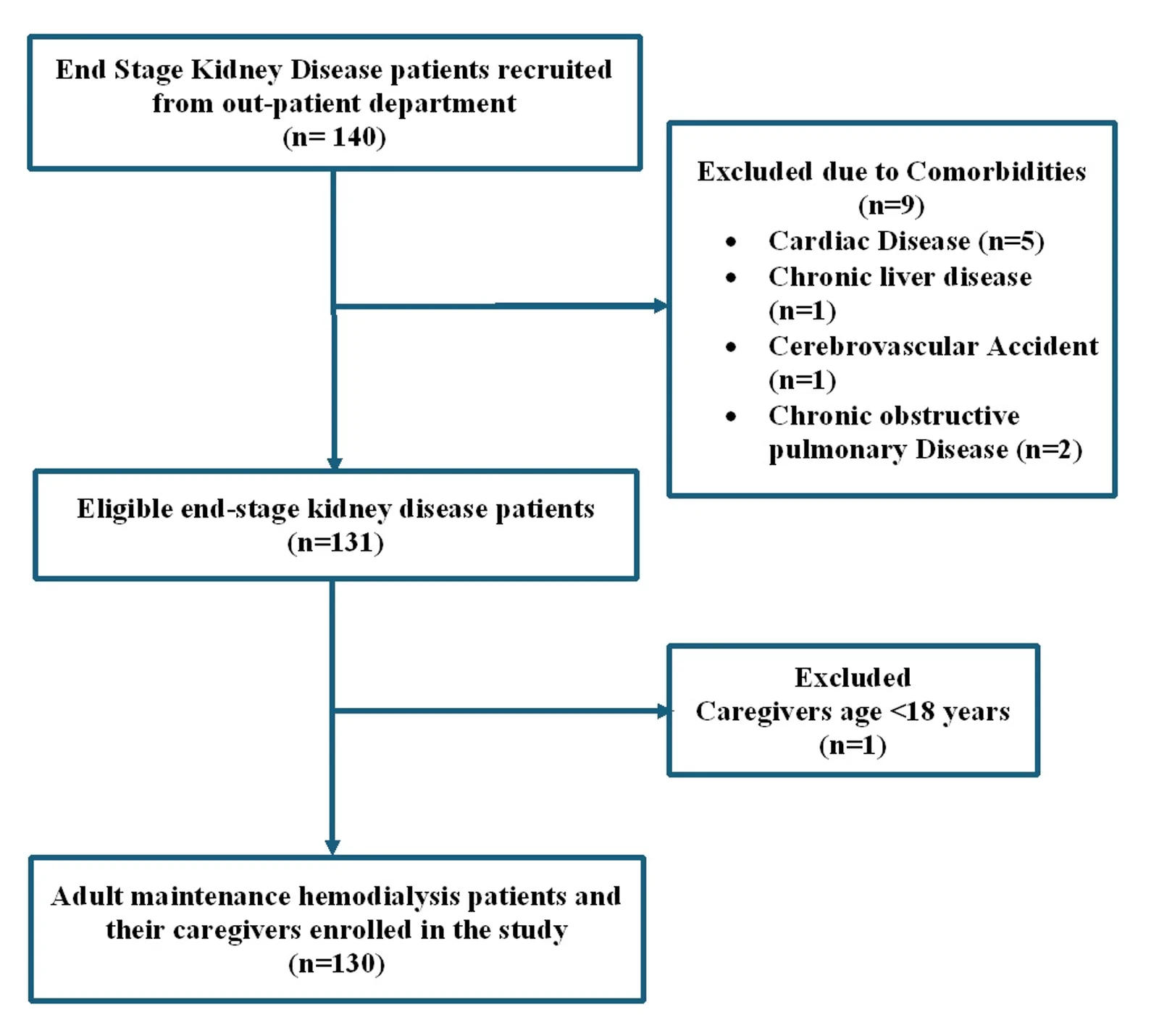
Participant flow diagram

Sociodemographic and clinical characteristics of patients and caregivers were recorded. Caregiver variables included age, gender, relationship with the patient, education, occupation, socioeconomic status by the modified Kuppuswamy scale (2025) [16], smoking history, comorbidities, distance from the dialysis unit, and average daily caregiving time. For analysis, lower socioeconomic status was defined as the combined lower and upper lower classes on the modified Kuppuswamy scale. Details of financial assistance from government-funded schemes or insurance claims were also recorded.

Caregiver burden was assessed using the ZBI-12 short-form, a validated 12-item questionnaire, yielding a total score of 0-48, with higher scores indicating greater burden [17]. Scores were categorized as 0-10 (no to mild burden), 11-20 (mild to moderate burden), and >20 (high burden) [10,17,18]. The ZBI (Hindi version) was used in this study with official permission from the copyright holders via Mapi Research Trust (MRT). 

The Institutional Ethics Committee of Motilal Nehru Medical College, Prayagraj, approved this study on 31st December 2023. Written informed consent was obtained from all participants, and the study was conducted in accordance with the ethical principles of the Declaration of Helsinki.

Statistical analysis

All statistical analyses were performed using IBM SPSS Statistics for Windows, Version 29 (Released 2022; IBM Corp., Armonk, New York, United States). Continuous variables were expressed as means with standard deviations or medians with interquartile ranges, as appropriate. Categorical variables were reported as frequencies and percentages. Logistic regression models were used to identify factors associated with high caregiver burden (ZBI-12 score >20); variables with p <0.20 in univariable analysis were entered into the multivariable model. All statistical tests were performed at a 5% level of significance, and p-values <0.05 were considered statistically significant.

## Results

This study enrolled 130 informal caregivers of hemodialysis patients. Sociodemographic characteristics of caregivers are presented in Table 1. The mean age of caregivers was 36.85 ± 12.98 years, and the majority were males (69.2%, n=90). Most lived in a nuclear family (n=87, 66.9%) and in an urban locality (n=93, 71.5%). Educational attainment was as follows: 45.4% (n=59) were graduates or had higher education, 22.3% (n=29) had higher-secondary education, and 9.2% (n=12) had secondary-level education. Most caregivers were productively engaged, either employed (52.3%, n=68) or homemakers (25.4%, n=33), while 13.8% (n=18) caregivers were students. The majority belonged to the lower-middle socioeconomic class (45.4%, n=59) on the modified Kuppuswamy scale (2025). Most caregivers were married (76.1%, n=99); spouses were the largest caregiver group (32.3%, n=42), followed by children and siblings. The mean caregiving time was 5.46 ± 1.52 hours per day, and the median distance to the dialysis center was 30 km (IQR, 8-60). Two caregivers had diabetes, and 10 (7.7%) had a history of smoking.

**Table 1:** Sociodemographic details of the caregivers (n=130) ^*^Modified Kuppuswamy socioeconomic scale (2025) [16] IQR: interquartile range; SD: standard deviation

Characteristic	Value
Age, years, mean ± SD	36.85 ± 12.98
Age group, years, n (%)	
18-30	50 (38.5%)
31-45	47 (36.2%)
46-60	26 (20.0%)
>60	7 (5.34%)
Male sex, n (%)	90 (69.2%)
Marital status, n (%)	
Married	99 (76.1%)
Unmarried	1 (0.8%)
Divorced	30 (23.1%)
Place of residence, n (%)	
Rural	37 (28.5%)
Urban	93 (71.5%)
Family type, n (%)	
Nuclear	87 (66.9%)
Joint	43 (33.1%)
Educational status, n (%)	
Illiterate	11 (8.5%)
Primary school	8 (6.2%)
Middle school	11 (8.5%)
Secondary school	12 (9.2%)
Higher secondary school	29 (22.3%)
Graduate or higher	59 (45.4%)
Occupational status, n (%)	
Employed	68 (52.3%)
Homemaker	33 (25.4%)
Student	18 (13.8%)
Unemployed	11 (8.5%)
Socioeconomic status^*^, n (%)	
Lower class (V)	9 (6.9%)
Upper-lower class (IV)	46 (35.4%)
Lower-middle class (III)	59 (45.4%)
Upper-middle class (II)	12 (9.2%)
Upper class (I)	4 (3.1%)
Relationship to patient, n (%)	
Parent	17 (13.1%)
Spouse	42 (32.3%)
Sibling	35 (26.9%)
Child	31 (23.8%)
Others	5 (3.8%)
Caregiving duration, hours/day, mean ± SD	5.46 ± 1.52
Distance to dialysis center, km, median (IQR)	30 (8-60)
History of smoking, n (%)	10 (7.7%)

Table 2 presents the baseline characteristics of the hemodialysis patients. The mean patient age was 42.88 ± 13.82 years, and 10% (n=13) were aged 60 years or older. Most patients were male (70%, n=91). Hypertension was the most common comorbidity (76.9%, n=100), followed by diabetes (23.1%, n=30) and hypothyroidism (9.2%, n=12). The median CKD duration was 19 months (IQR, 7-33.2), and the median dialysis vintage was 10 months (IQR, 6-20). Patients underwent a mean of 2.08 ± 0.26 dialysis sessions per week. Overall, 24.6% (n=32) of patients were not engaged in productive work, and 46.2% (n=60) received financial assistance for treatment.

**Table 2:** Baseline characteristics of the hemodialysis patients (n=130) CKD: chronic kidney disease; IQR: interquartile range; SD: standard deviation

Characteristic	Value
Age, years, mean ± SD	42.88 ± 13.82
Elderly patients (age ≥60 years), n (%)	13 (10.0%)
Male sex, n (%)	91 (70.0%)
Comorbidities, n (%)	
Diabetes	30 (23.1%)
Hypertension	100 (76.9%)
Hypothyroidism	12 (9.2%)
CKD duration, months, median (IQR)	19 (7-33.2)
Dialysis vintage, months, median (IQR)	10 (6-20)
Dialysis sessions/week, mean ± SD	2.08 ± 0.26
Occupational status, n (%)	
Unemployed	32 (24.6%)
Homemaker	27 (20.8%)
Employed	71 (54.6%)
Financial aid received, n (%)	60 (46.2%)

The mean ZBI-12 score was 22.32 ± 7.21 (Range 7-36). High caregiver burden, defined as a score >20, was observed in 77 caregivers (59.2%); 40 (30.8%) had mild-to-moderate burden (scores 11-20), and 13 (10.0%) had no-to-mild burden (scores 0-10) (Table 3). In multivariable analysis, lower socioeconomic status was the only variable independently associated with high caregiver burden (adjusted OR 2.90, 95% CI 1.29-6.52; p=0.010) (Table 4).

**Table 3:** Distribution of caregiver burden levels measured by ZBI-12 (n=130) SD: standard deviation; ZBI: Zarit Burden Interview

ZBI-12 outcome	Value
Mean ± SD	22.32 ± 7.21
No to mild burden (score 0–10), n (%)	13 (10.0%)
Mild to moderate burden (score 11–20), n (%)	40 (30.8%)
High burden (score >20), n (%)	77 (59.2%)

**Table 4:** Factors associated with high caregiver burden (ZBI-12 score >20) among informal caregivers of hemodialysis patients *Lower socioeconomic status represents combined lower and upper-lower classes on the modified Kuppuswamy scale (2025) Variables with p <0.20 in univariable analysis were entered into the multivariable model OR: odds ratio

Variables	Unadjusted OR (95% CI)	p-value	Adjusted OR (95% CI)	p-value
Age of the caregivers	0.99 (0.97-1.02)	0.785	-	-
Elderly patient (age ≥60 years)	0.34 (0.10-1.07)	0.067	0.34 (0.10-1.15)	0.085
Female caregivers	0.77 (0.36-1.65)	0.513	-	-
Caregivers with ≥ secondary school education	0.54 (0.22-1.30)	0.175	0.81 (0.30-2.16)	0.678
Employed caregivers	1.09 (0.54-2.20)	0.796	-	-
Lower socioeconomic status*	3.17 (1.48-6.76)	0.003	2.90 (1.29-6.52)	0.010
Caregiving duration per day	0.91 (0.72-1.15)	0.444	-	-
Distance from dialysis unit	1.00 (0.99-1.01)	0.492	-	-
Dialysis Vintage	0.99 (0.96-1.01)	0.474	-	-
Lack of financial aid	0.49 (0.23-1.00)	0.052	0.48 (0.22-1.03)	0.060

## Discussion

Caregivers play an essential role in the management of CKD, particularly once patients are initiated on maintenance hemodialysis [6]. The impact of ESKD extends beyond the patient and affects the entire family. Hemodialysis imposes direct and indirect costs, including medications, transportation, and lost employment opportunities. Family members are also exposed to the emotional trauma of witnessing their loved ones with a life-threatening illness, with added uncertainties about their long-term health outcomes. Caregivers frequently reschedule their personal and professional commitments to accommodate the patient’s dialysis days, hospital appointments, and personal needs. This multifactorial caregiver burden stems from the persistent psychological distress due to concerns about the patient’s health, needs of dialysis, and financial constraints, ultimately affecting their overall quality of life [19,20].

This study found a significant burden among informal caregivers of maintenance hemodialysis patients at a tertiary care center in Uttar Pradesh, India, consistent with findings from previous Indian studies summarized in Table 5. The mean ZBI-12 score was 22.32 ± 7.21, and 59.2% reported a high caregiver burden. In comparison, Ramasamy et al., who also used the ZBI-12, found a lower mean score of 18.55 ± 7.58, with most caregivers (59%) experiencing mild to moderate caregiver burden [10]. This difference may be due to variations in the clinical and socioeconomic characteristics of the study populations, caregiving demands, assessment tools, and diagnostic thresholds used to classify caregiver burden across studies.

**Table 5:** Summary of studies on caregiver burden among caregivers of hemodialysis patients and their predictors/associated factors Burden categories should be interpreted cautiously because the studies used different instruments, score thresholds, and category labels. *Lower socioeconomic status represents combined lower and upper-lower classes on the modified Kuppuswamy scale (2025); ^#^Majority of patients (39%) were in the 20-39-year age group CBI: caregiver burden inventory; SD: standard deviation; ZBI: Zarit Burden Interview

Study	Setting	Sample size, n	Caregiver age, years, Mean ± SD	Female caregivers, n (%)	Instrument	Burden distribution	Reported predictors/ associated factors
Present study	Prayagraj, India	130	36.85 ± 12.98	40 (30.8%)	ZBI	No to mild - 10%, Mild to moderate - 30.8%, High - 59.2%	Lower socioeconomic status^*^
Rafati et al. [11]	Kerman, Iran	382	42.9 ± 13.9	182 (48%)	CBI	Mild - 11.8%, Moderate - 56%, High - 32.2%	Patient’s income, dialysis frequency, care needs, spiritual well-being
Singh et al. [13]	Delhi, India	310	39.69 ± 12.68	115 (37%)	CBI	Low - 36.5%, Moderate - 55.5%, Severe - 8.0%	Planning coping strategies, psychological quality of life, social support, employment, retirement status
Azeez et al. [9]	Thiruvananthapuram, India	200	55 ± 12.53	155 (77.5%)	ZBI	Little to no burden - 42%, Mild to moderate - 46%, Moderate to severe - 11%, Severe - 1%	Financial stress, lack of leisure time, caregiver comorbidity
Chhetri et al. [12]	Chitwan, Nepal	123	NA^#^	71 (58%)	ZBI	Little to no burden - 4.8%, Mild to moderate - 48.7%, Moderate to severe - 43%, Severe - 3.2%	NA
Ramasamy et al. [10]	Raipur, India	105	40.0 ± 12.9	56 (53%)	ZBI	Minimal - 12.4%, Mild to moderate - 59%, High - 28.6%	NA
Arshad et al. [15]	Rawalpindi, Pakistan	98	33.13 ± 9.98	27 (27.6%)	ZBI	Little to no burden - 27.6%, Mild to moderate - 53.1%, Moderate to severe - 18.4%, Severe - 3.1%	Female caregiver

Caregivers in our cohort were relatively young and predominantly male, which is consistent with previous studies [13,21]. This may be because younger family members are more likely to take an active role in accompanying patients for dialysis and managing their treatment-related needs. The observed male predominance reflects regional social and cultural norms, in which men typically assume responsibility for finances, transportation, and hospital visits. However, Rafati et al. and Azeez et al. reported a mean caregiver age above 40 years [9,11], and Ramasamy et al. and Chhetri et al. documented females as the predominant caregiving group [10,12]. Arshad et al. and Joseph et al. reported severe caregiver burden among female and older caregivers [15,22], but neither age nor sex was associated with high caregiver burden in our cohort.

Lower socioeconomic status was the only factor independently associated with high caregiver burden in our study (adjusted OR 2.90, 95% CI 1.29-6.52; p=0.010). This may be related to poverty, social and educational disadvantages, and limited access to healthcare facilities. Socioeconomic status is influenced by educational level, occupation of the head of the household, and monthly family income, with financial strain being an important factor in caregiver burden. Previous studies have also reported an association between financial difficulties and caregiver burden. Bahall et al. found that 68% of caregivers in their cohort experienced economic hardship and financial worries [23]. The high and lifelong cost of dialysis may place considerable pressure on family finances, making it difficult for economically disadvantaged households to manage both the financial demands of treatment and the responsibilities of long-term caregiving.

Financial subsidies, such as the Ayushman Bharat scheme and the Chief Minister’s Relief Fund, are provided by the government to support eligible families with healthcare expenses. In our cohort, lack of financial aid was not significantly associated with high caregiver burden. This unexpected finding may reflect differences in the socioeconomic and clinical characteristics of families receiving financial assistance, as such support may be more commonly sought by families with greater financial difficulties. After adjustment for other factors, particularly socioeconomic status, the association was no longer observed. Therefore, this finding should be interpreted cautiously, and it does not suggest that the absence of financial aid reduces caregiver burden.

Although elderly patients may have greater physical dependence and require more assistance with daily activities, caring for an elderly patient was not associated with high caregiver burden in our cohort. Caregivers of younger patients may experience greater psychological and emotional stress because of concerns about their long-term health, future, and disruption of their personal, professional, and social lives. In contrast, caregiving for older patients may be perceived differently because of differences in expectations regarding life expectancy and the course of illness. The lack of a significant association may also be related to the small number of elderly patients in our cohort, which may have limited the statistical power to detect an association.

Azeez et al. have reported that caregivers without leisure time had increased odds of caregiver burden [9]. Similarly, Farzi et al. reported that daily caregiving hours were significantly related to caregivers’ quality of life [24]. These findings suggest that the intensity of caregiving and the limited opportunity for leisure activity may negatively affect caregivers' well-being. However, in contrast to these studies, our study found no significant association between the duration of caregiving and caregiver burden. This may be due to the development of coping strategies over time, family support, and sharing of caregiving responsibilities.

In our study, patient characteristics like age, gender, and dialysis vintage were not associated with caregiver burden, although some studies have reported a relationship between patient illness severity and caregiver stress [14]. This can be explained by the multifactorial etiology of caregiver burden, determined by the patient’s condition, caregiver characteristics, and social support, rather than age, gender, or duration of dialysis alone. Although longer dialysis vintage may increase caregiving needs, caregivers may gradually adapt to the treatment routine and develop effective coping strategies over time. Furthermore, dialysis vintage may not reflect the patient's functional dependence or illness severity. Differences in family-friend support systems, financial conditions, coping mechanisms, and cultural expectations may also contribute to the variations observed in previous studies.

The strengths of this study include the assessment of both caregiver and patient characteristics, use of a validated brief instrument to measure caregiver burden, and multivariable regression to evaluate factors independently associated with high caregiver burden. Nevertheless, several limitations should be considered. The sample size was small (n=130), and the study was conducted at a single center, which may limit the generalizability of the findings. Caregiving time and financial support were self-reported and may be affected by recall or reporting bias. Because the study was cross-sectional, temporal relationships and causality cannot be established. Coping mechanisms and social support were not assessed and may have contributed to residual confounding. In addition, excluding hemodialysis patients with major comorbid conditions requiring additional care may limit applicability to the broader hemodialysis population and may have resulted in a lower estimate of real-world caregiver burden. 

## Conclusions

Caregiver burden was common among informal caregivers of patients receiving maintenance hemodialysis in this single-center study, and lower socioeconomic status was independently associated with high caregiver burden. These findings support consideration of routine caregiver screening, education, counseling, and targeted socioeconomic and psychosocial support within nephrology services, particularly for resource-constrained households. Longitudinal and interventional studies are needed to determine whether such strategies improve caregiver well-being or patient outcomes.

## References

[1] GBD 2023 Chronic Kidney Disease Collaborators (2025). Global, regional, and national burden of chronic kidney disease in adults, 1990-2023, and its attributable risk factors: a systematic analysis for the Global Burden of Disease Study 2023. Lancet.

[2] Cojuc-Konigsberg G, Guijosa A, Moscona-Nissan A (2025). Representation of low- and middle-income countries in CKD drug trials: a systematic review. Am J Kidney Dis.

[3] Prasad N, Meyyappan J, Veeranki V (2024). Nephrology services in Uttar Pradesh. IJN.

[4] Liu Z, Heffernan C, Tan J (2020). Caregiver burden: a concept analysis. Int J Nurs Sci.

[5] Sharpe L, Butow P, Smith C, McConnell D, Clarke S (2005). The relationship between available support, unmet needs and caregiver burden in patients with advanced cancer and their carers. Psycho Oncol.

[6] Hoang VL, Green T, Bonner A (2018). Informal caregivers' experiences of caring for people receiving dialysis: a mixed-methods systematic review. J Ren Care.

[7] Zarit SH, Reever KE, Bach-Peterson J (1980). Relatives of the impaired elderly: correlates of feelings of burden. The Gerontologist.

[8] Nagarathnam M, Latheef SA, Sivakumar V (2021). Factors influencing scales of burden, coping mechanisms, and quality of life in caregivers of hemodialysis patients in Andhra Pradesh, India. Indian J Palliat Care.

[9] Azeez A, Ambatipudi S (2024). Caregiver burden and quality of life among family caregivers of hemodialysis patients from South India. J Educ Health Promot.

[10] Ramasamy S, Rathore V, Agrawal V, Galhotra A (2025). Burden level and quality of life of caregivers of hemodialysis patient's in central India: a mixed-method cross-sectional study. BMC Res Notes.

[11] Rafati F, Mashayekhi F, Dastyar N (2020). Caregiver burden and spiritual well-being in caregivers of hemodialysis patients. J Relig Health.

[12] Chhetri SK, Baral R (2020). Caregiver burden among caregivers of patient undergoing hemodialysis in tertiary care center: a descriptive cross-sectional study. JNMA J Nepal Med Assoc.

[13] Singh NP, Kaul S, Khullar D, Gupta AK, Sharma S, Sarkar T.Caregiver Burden (2026). Quality of life, and coping strategies among caregivers of patients undergoing maintenance hemodialysis: a cross-sectional study. IJN.

[14] Jafari H, Ebrahimi A, Aghaei A, Khatony A (2018). The relationship between care burden and quality of life in caregivers of hemodialysis patients. BMC Nephrol.

[15] Arshad AR, Tahir T, Mir AW, Salahuddin (2021). Psychological burden amongst caregivers of patients on maintenance haemodialysis. J Coll Physicians Surg Pak.

[16] Mandal I, Hossain SR (2025). Modified Kuppuswamy scale updated for the year 2025. Int J Community Med Public Health.

[17] Bédard M, Molloy DW, Squire L, Dubois S, Lever JA, O'Donnell M (2001). The Zarit Burden Interview: a new short version and screening version. Gerontologist.

[18] Yu J, Yap P, Liew TM (2019). The optimal short version of the Zarit Burden Interview for dementia caregivers: diagnostic utility and externally validated cutoffs. Aging Ment Health.

[19] Driehuis E, Demirhan I, Konijn WS (2025). Determinants of caregiver burden among spouses of patients with kidney failure: a qualitative study. Am J Kidney Dis.

[20] Low J, Smith G, Burns A, Jones L (2008). The impact of end-stage kidney disease (ESKD) on close persons: a literature review. NDT Plus.

[21] Sharma M, Lakhara P, Sharma SK, Jelly P, Sharma R (2021). The burden of caregivers of patients undergoing hemodialysis. J Holist Nurs Midwifery.

[22] Joseph SJ, Bhandari SS, Dutta S, Khatri D, Upadhyay A (2021). Assessing burden and its determinants in caregivers of chronic kidney disease patients undergoing haemodialysis. Open J Psychiatry Allied Sci.

[23] Bahall M, Harry AD, Kisseur A (2025). Self-reported socio-economic and psychological burdens for caregivers of patients undergoing dialysis: a cross-sectional study. Cureus.

[24] Farzi S, Farzi S, Moladoost A, Ehsani M, Shahriari M, Moieni M (2019). Caring burden and quality of life of family caregivers in patients undergoing hemodialysis: a descriptive-analytic study. Int J Community Based Nurs Midwifery.

